# Plasma 25-Hydroxyvitamin D and Its Genetic Determinants in Relation to Incident Myocardial Infarction and Stroke in the European Prospective Investigation into Cancer and Nutrition (EPIC)-Germany Study

**DOI:** 10.1371/journal.pone.0069080

**Published:** 2013-07-25

**Authors:** Tilman Kühn, Rudolf Kaaks, Birgit Teucher, Frank Hirche, Jutta Dierkes, Cornelia Weikert, Verena Katzke, Heiner Boeing, Gabriele I. Stangl, Brian Buijsse

**Affiliations:** 1 Division of Cancer Epidemiology, German Cancer Research Center, Heidelberg, Germany; 2 Institute of Agricultural and Nutritional Sciences, Martin-Luther-University Halle-Wittenberg, Halle (Saale), Germany; 3 Department of Clinical Medicine, University of Bergen, Bergen, Norway; 4 Department of Epidemiology, German Institute of Human Nutrition Potsdam-Rehbrücke, Nuthetal, Germany; German Diabetes Center, Leibniz Center for Diabetes Research at Heinrich Heine University Duesseldorf, Germany

## Abstract

Circulating 25-hydroxyvitamin D (25(OH)D) has been associated with cardiovascular disease (CVD) risk in observational studies. Also, SNPs to explain variation in 25(OH)D have been identified by genome-wide association studies. Detection of direct associations between SNPs that significantly affect 25(OH)D and CVD risk would indicate a causal role of vitamin D, as reverse causation could be excluded and confounding could be better controlled. Thus, a combined analysis of candidate SNPs in relation to circulating 25(OH)D and CVD risk was carried out. A case-cohort study within the EPIC-Germany study was conducted comprising a randomly drawn subcohort of 2,132 subjects (57.9% women, mean age: 50.6 years) and incident cases of myocardial infarction (n=559) and stroke (n=471) that occurred during a mean follow-up duration of 7.6 years. 25(OH)D concentrations were measured by LC-MS/MS in baseline plasma samples. Additionally, eight candidate SNPs were assayed. Associations between 25(OH)D, SNPs and the risks of myocardial infarction and stroke were assessed by multivariable regression analyses. Mean 25(OH)D level was 47.2 nmol/L in the subcohort. Four SNPs were associated with 25(OH)D (p<0.05). In subjects with 25(OH)D levels <25 nmol/L, the risks of CVD as composite endpoint (Hazard Ratio: 1.53, 95% confidence interval: 1.12–2.09), myocardial infarction, and stroke were significantly increased compared to subjects with levels ≥50 nmol/L, while no significant linear associations were observed. A SNP score was not related to the risks of total CVD (Hazard Ratio: 1.0, 95% confidence interval: 0.71–1.42), myocardial infarction, or stroke. The same was true concerning single SNPs. Given the lack of association between SNPs and the risks of stroke and myocardial infarction, the present findings do not point to a major causal role of vitamin D in the development of these diseases. However, a detection of modest associations between genetic markers and CVD risk in larger consortia cannot be ruled out.

## Introduction

Beyond its role in bone health, vitamin D has been proposed to lower the risks of a variety of non-skeletal health outcomes including cardiovascular diseases (CVD) [1,2]. Its active form, 1,25(OH)_2_vitamin D, is a secosteroid hormone whose synthesis starts by a UV–B induced conversion of 7-dehydrocholesterol to cholecalciferol (vitamin D_3_) in the human skin [3]. In addition, dietary cholecalciferol from animal food sources as well as dietary ergocalciferol (vitamin D_2_) from mushrooms contribute to vitamin D supply, even though to a lesser extent than cholecalciferol from cutaneous production [2,4]. In the circulation, vitamin D_2_ and D_3_ are transported to the liver bound to the vitamin D binding protein and converted to 25-hydroxyvitamin D (25(OH)D) by the vitamin D 25-hydroxylase. 25(OH)D, the accepted clinical indicator of vitamin D status, is activated to 1,25(OH)_2_vitamin D by the vitamin D 1-alpha-hydroxylase in the kidneys and, in a paracrine fashion, in several extra-renal tissues. Biological actions of activated vitamin D in the target organs are mediated through the vitamin D receptor (VDR) [3].

The detection of the VDR and the 1-alpha-hydroxylase in cells throughout the cardiovascular system has motivated multiple studies on the role of vitamin D in the etiology of CVD [5,6]. Several pathways linking vitamin D status with atherogenesis and CVD risk have been identified in experimental studies. Vitamin D seems to down-regulate the renin-angiotensin aldosterone system (RAAS), inflammation, coagulation, proliferation of vascular smooth muscle cells and cardiomyocytes, vascular calcification, and parathyroid hormone levels [5-8]. At the same time, vitamin D may improve insulin secretion and insulin sensitivity, as well as endothelial function [6,8].

CVD risk may be lowered by these mechanisms and pooled results from observational studies actually also suggest that higher circulating 25(OH)D levels are associated with decreased risks of stroke [9] and myocardial infarction [10]. However, it has been challenged that vitamin D is causally linked with the development of CVD [11]. Instead, low circulating 25(OH)D levels might be a surrogate for a poor overall health status, rather reflecting the inability to get outdoors because of multimorbidity, low exercise resistance, or increased BMI [12].

Although large randomized controlled trials (RCT) to elucidate the effects of vitamin D supplement use on CVD risk are under way, results are not to be expected before 2017 [13]. Also, concerns have already been raised about compliance, statistical power, latitude of the study centers, target populations, or the applied vitamin D dosages in these trials [13-15]. As a complementary approach to RCTs, observational studies with combined analyses of circulating 25(OH)D and its genetic determinants in relation to CVD endpoints may help to assess, whether the link between vitamin D status and CVD risk is causal [14,16]. Several single nucleotide polymorphisms (SNPs) coding for the vitamin D binding protein and enzymes involved in vitamin D synthesis and metabolism have been found to be associated with vitamin D status in genome wide association studies (GWAS) [17,18]. Concurrent associations of such SNPs with both vitamin D status and CVD risk in observational studies would indicate a causal link between vitamin D status and CVD risk, as genetic variation is assigned randomly during meiosis and reverse causation could be ruled out [16,19].

To date, only one study has assessed the association between vitamin D related SNPs, vitamin D status and the risk of myocardial infarction (MI) [20], while no comparable study on stroke risk has been carried out. Therefore, we investigated the associations between genetic vitamin D status determinants, circulating 25(OH)D, and the risks of MI and stroke in the German branch of the European Prospective Investigation into Cancer and Nutrition (EPIC). 

## Methods

### Population and study design

The European Investigation into Cancer and Nutrition (EPIC) is an ongoing prospective study of chronic diseases in 10 European countries. In Germany, it comprises two cohorts at centers in Heidelberg and Potsdam [21]. Between 1994 and 1998, 25 440 participants (13 612 women and 11 928 men) were recruited in Heidelberg and 27 548 participants (16 644 women and 10 904 men) in Potsdam. Subjects were mostly aged between 35 and 65 years at both centers. Baseline examinations included detailed self-administered questionnaires and interviews on health status, socio-economic status and lifestyle, anthropometric measurements, and a dietary assessment by food frequency questionnaire [21]. A blood sample from about 95% of the participants was drawn, processed and stored at -196° Celsius in liquid nitrogen [21]. Participants are being followed-up by active and passive procedures [22].

**Figure 1:**
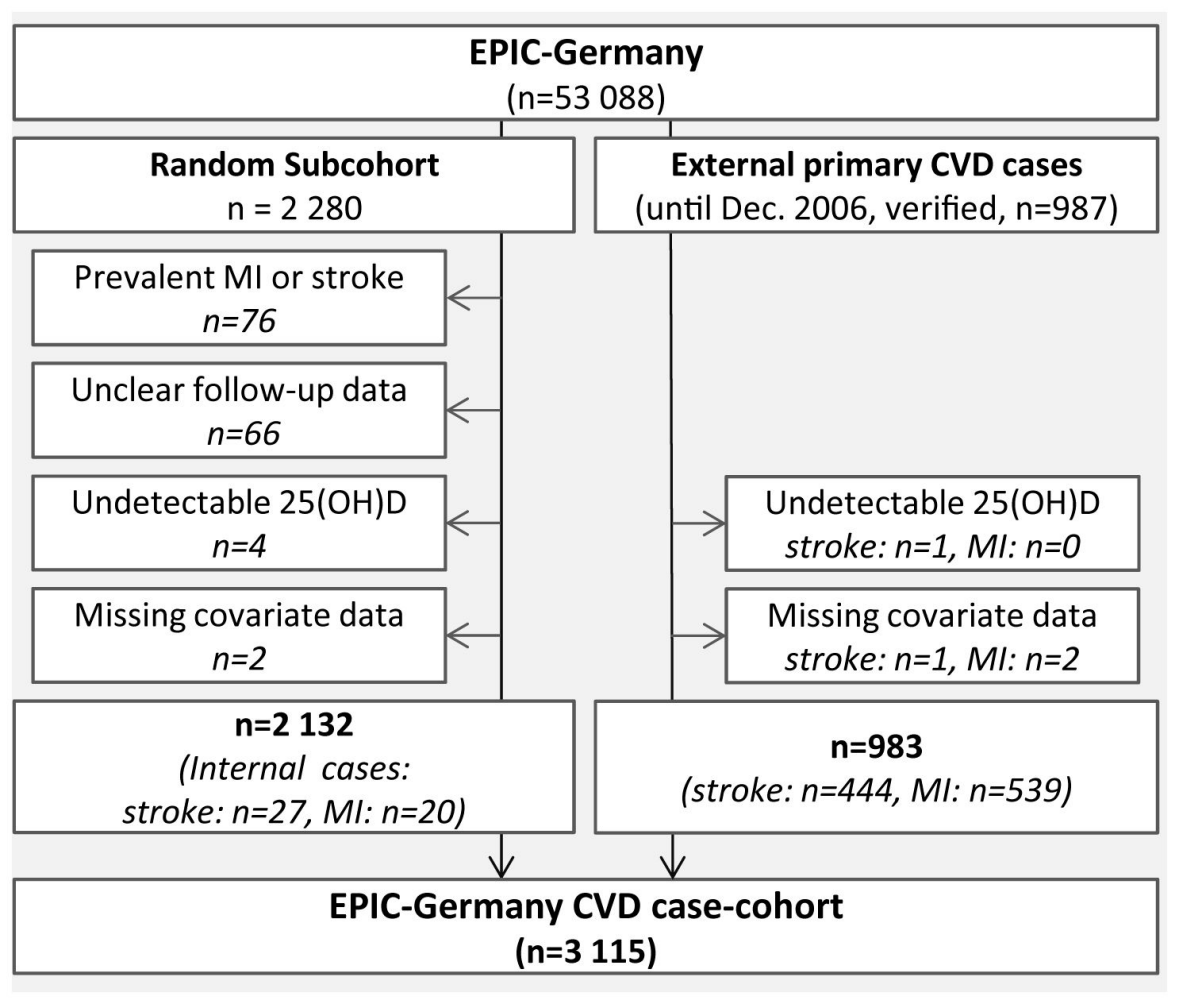
Flow chart indicating exclusions due to prevalent CVD and missing values.

A case-cohort design was chosen for the analyses on 25(OH)D in relation to CVD risk [23,24]. Random subcohorts were drawn in Heidelberg and Potsdam according to the protocol of the Europe-wide EPIC InterAct study on diabetes type 2 [25]. As opposed to the InterAct selection criteria, prevalent cases of diabetes mellitus type 2 were kept in the present study. After the exclusion of participants with prevalent MI or stroke (n=76), without complete follow-up information (n=66), missing 25(OH)D values (n=4), and missing covariate data (n=2), the common subcohort consisted of 2 132 participants (Figure 1). In addition, incident cases of primary MI and stroke from complete follow-up rounds up to the end of December 2006 were included into the case-cohort study. MIs and strokes were ascertained by study physicians who carried out a detailed and systematic medical verification of self-reports and death certificates by clinical records in cooperation with treating physicians and hospitals. Outcomes were classified as fatal in case of death within the first 28 days after diagnosis. The following conditions were coded according to the International Statistical Classification of Diseases, 10th revision, 2011 (ICD-10): MI (ICD-10 I21), ischemic stroke (ICD-10 I63), hemorrhagic stroke (ICD-10 I60 and I61) and unspecified stroke (ICD-10 I64). After exclusion of incident cases with undetectable 25(OH)D levels (n_MI_=1) or missing covariate data (n_MI_=1, n_stroke_=2), 559 verified cases of MI and 471 verified cases of stroke that had occurred during average follow-up periods of 7.5 (±2.3) and 7.7 (±2.1) years, respectively, were included into the CVD case-cohort study (Figure 1). Out of these, 20 MI cases and 27 stroke cases were part of the random subcohort. 90 (16.1%) MI cases and 50 (10.6%) stroke cases were fatal. 363 (77.1%) stroke cases were classified as ischemic, 88 (18.7%) as hemorrhagic, and 20 (4.2%) cases as unspecified.

### Ethics statement

The study was approved by ethics committees at both study sites (Potsdam: Ethics Committee of the Medical Association of the State of Brandenburg; Heidelberg: Ethics Committee of the Heidelberg University Medical School) and all participants gave written informed consent.

### Laboratory methods

Vitamin D status was determined in the laboratory of the Institute of Agricultural and Nutritional Sciences at the University of Halle-Wittenberg, Germany. 25(OH)D_2_ and 25(OH)D_3_ were assayed in plasma samples from baseline that had been stored at -196° Celsius for about 15 years on average. A MassChrom^®^ reagent kit (Chromsystems, Munich, Germany) was used on an LC/MS-MS system (API 2000™, Applied Biosystems, Darmstadt, Germany) to measure both metabolites. Samples of incident cases and subcohort members were assigned to 78 analytical batches. Each batch contained at least 30 samples from both study centers and included both case and control samples. Laboratory staff was blinded to the case status of the analyzed samples. The within-batch coefficients of variation for 25(OH)D_2_ and 25(OH)D_3_ were 4.9% at 43.5 nmol/L and 4.2% at 96.8 nmol/L (n=3), and 5.6% at 41.8 nmol/L and 3.5% at 95.8 nmol/L (n=3), respectively. Between-batch coefficients of variation for 25(OH)D_2_ and 25(OH)D_3_ and were 5.8% at 43.5 nmol/L and 5.6% at 96.8 nmol/L, as well as 7.8% at 41.8 nmol/L and 6.6% at 95.8 nmol/L. The use of quality control samples from the Vitamin D External Quality Assessment Scheme (www.deqas.org) revealed that 80% of the results fell within ±15% of the ALTM (All Laboratory Trimmed Mean), as compared to ±25% being the goal. The lower limit of quantification (LLOQ) was 8.25 nmol/L for 25(OH)D_2_ and 11.5 nmol/L for 25(OH)D_3,_ the lower limit of detection (LLOD) was 2.75 nmol/l for 25(OH)D_2_ and 4.5 nmol/L for 25(OH)D_3_. For measured values above the LOD, but below the LLOQ, values equal to half of the LLOQ were assigned for statistical analyses. Overall, 25 out of 3 115 detected 25(OH)D_3_ values, and 127 out of 174 detected 25(OH)D_2_ values were below the LLOQ.

Candidate SNPs were selected based on the results of two recent GWAS [17,18] and a systematic review [26]. Information regarding linkage disequilibrium (LD) and minor-allele frequencies (MAF) of candidate SNPs was obtained using HapMap data (database release 27, CEU and TSI populations). SNPs characterized by a MAF <0.15 or a high LD with other candidates (R^2^≥0.8) were not considered. Overall 8 SNPs from 6 regions were selected: rs1155563 and rs2282679 in the *GC* (group specific component) locus that codes for the vitamin D binding protein; rs3829251 and rs12785878 in the *DHCR7/NADSYN1* locus that codes for the 7-dehydrocholesterol reductase involved in converting 7-dehyrocholestrol, the substrate for cholecalciferol, to cholesterol; rs10741657 in the *CYP2R1* locus (25-hydroxylase); rs10877012 in the *CYP27B1* locus (1-alpha-hydroxylase); rs6013897 in the *CYP24A1* locus (24-hydroxylase that deactivates 1,25(OH)_2_vitamin D); rs6599638 in the *C10orf88* locus in the vicinity of the gene coding for the acyl-Coenzyme A dehydrogenase, an enzyme involved in cholesterol synthesis;

Buffy-coat samples retrieved from liquid nitrogen storage were used for DNA extraction. Genotyping was carried out at KBioscience (Hoddesdon, Herts, UK) using the KASP assay, a homogeneous fluorescent genotyping system based on competitive allele-specific PCR. For quality control purposes non-template controls (NTC) were included on each plate. Genotyping call rates exceeded 96% for each SNP.

### Statistical evaluation

It was decided to use total 25(OH)D values for the statistical analyses, because 25(OH)D_2_ was detected in 5.6% of the samples only. To standardize 25(OH)D levels for season of blood draw, residuals obtained from locally weighted polynomial regression of 25(OH)D on calendar week of blood draw were added to the overall case-cohort mean value [27,28].

Hazard ratios (HR) of MI and stroke were calculated with Cox proportional-hazards models modified by the Prentice method to account for the case-cohort design of the study [23,24]. 25(OH)D levels were grouped into quartiles setting the highest quartile as the reference. In addition, we used clinical categories of 25(OH)D levels as defined by the Robert Koch Institute (severe to moderate deficiency: <25 nmol/L, mild deficiency: 25-49.9 nmol/L, sufficiency: ≥50 nmol/L) as predictors in our models [29]. Age was used as the underlying timescale. The baseline hazard function was allowed to differ by age (1 year integers) and center. First, a crude model was built adjusting for sex and BMI (continuous). Further potential confounders identified by literature search, i.e. waist circumference (cm, continuous), smoking (‘never’, ‘former’, ‘1-20 cigarettes per day’, ‘>20 cigarettes per day’), physical activity according to the Cambridge Physical Activity Index [30] (‘active’, ‘moderately active’, ‘moderately inactive’, ‘inactive’), and alcohol consumption (g/d, continuous), as well as educational attainment as proxy for the subjects’ socioeconomic status (‘low’: primary school or no school graduation; ‘medium’: secondary, technical or professional school graduation; ‘high’: longer education including university degree) were additionally included in the second model. Linear trends were tested treating quartile medians as quantitative exposure scores. Besides the analyses with season-standardized 25(OH)D values, regression models were recalculated using quartiles of raw 25(OH)D values with adjustment for month of blood draw or season-specific 25(OH)D quartiles.

To check for non-linearity, Cox models with first and second degree fractional polynomials of 25(OH)D levels were applied allowing for seven transformations of continuous 25(OH)D values (by x^-2^, x^-1^, x^-0.5^, x^0.5^, x^2^, x^3^, and log(x)) [31]. 25(OH)D values below the lower limit of quantification and in the highest percentile of the distribution were omitted from fractional polynomial analyses. The fractional polynomial with the best fit according to deviance testing was selected [31]. Log likelihood ratio tests were conducted to detect interactions between 25(OH)D levels with adjustment variables. Sensitivity analyses were carried out excluding the cases that occurred within the first two years after baseline.

Correlation coefficients and Lewontin’s D' were calculated as measures for the Linkage disequilibrium (LD) between SNP pairs. Chi-squared tests were conducted to assess if genotypes were in Hardy-Weinberg equilibrium (HWE). Generalized Linear Models were used to assess the association between SNPs and 25(OH)D in the subcohort adjusting for age, sex and study center. P values were multiplied by 8 to account for multiple testing. Partial R^2^ values were calculated as estimates for the effect sizes of the SNPs. HRs of MI and stroke across alleles were calculated by Cox proportional-hazards models adjusted for sex and center using age as the underlying timescale. Co-dominant, dominant, log-additive, and recessive models were used for analyses on single SNPs. Further, risk allele scores were calculated based on SNPs that were significantly associated with 25(OH)D levels. First, a simple score was created summing the number of alleles associated with lower 25(OH)D levels. Alternatively, risk alleles were weighted by beta coefficients obtained from the regression of SNPs on 25(OH)D levels using an additive model. Statistical tests were two-sided and p-values of <0.05 were considered statistically significant. SAS 9.2 (SAS Institute, NC, USA) was used for all analyses.

## Results

### Characteristics of the study participants

**Figure 2:**
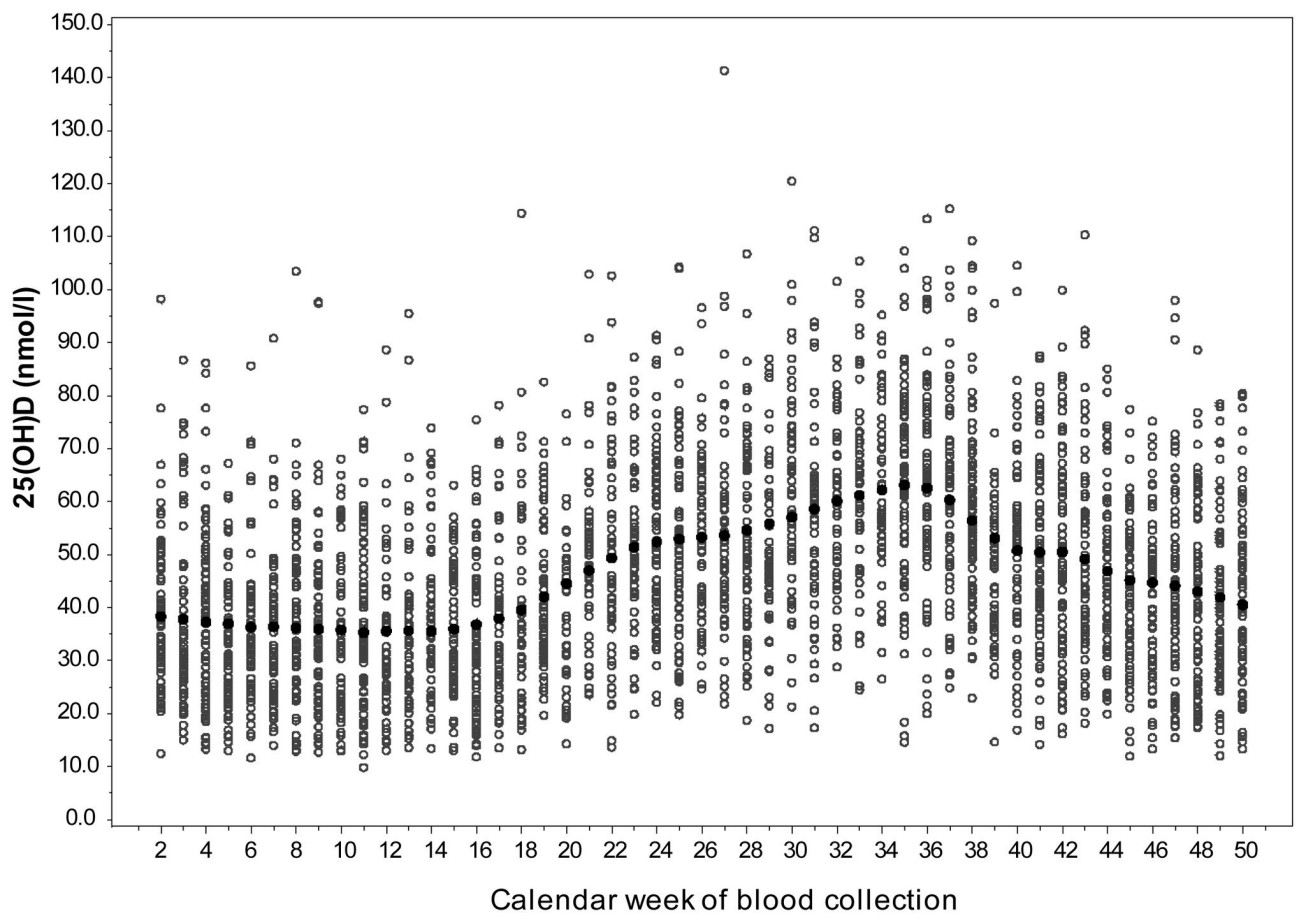
Seasonal variation in plasma 25(OH)D levels. The light circles represent individual 25(OH)D plasma concentrations, the black circles represent weekly average 25(OH)D plasma concentrations after smoothing by locally weighted polynomial regression.

The seasonal variation in 25(OH)D levels in the subcohort is shown in Figure 2. Mean season-adjusted 25(OH)D levels were higher among non-cases in the subcohort (47.3 nmol/L, ±18.3) and in the total subcohort (47.2 nmol/L, ±18.3) when compared to the mean levels in MI cases (44.6 nmol/L, ±18.9) and stroke cases (44.6 nmol/L, ±18.3). Further baseline characteristics of the subcohort and the incident cases are presented in Table 1. Incident MI and stroke cases were older and more likely to be men compared to the subcohort members. They had higher risk profiles concerning classical CVD risk factors including overweight and obesity, smoking, physical activity, hypertension, and diabetes. Also, CVD cases had higher waist circumference values and lower formal education levels. Finally, average alcohol intake levels in stroke cases were higher than in subcohort members.

**Table 1:** Characteristics of the subcohort presented by quartiles of season-standardized 25(OH)D.

	Subcohort* (quartiles of season-standardized plasma 25(OH)D)						
	**Q1**	**Q2**	**Q3**	**Q4**	**All**	**MI cases**	**stroke cases**
N	533	533	533	533	2132	559	471
Women	62.5	58.5	55.4	55.2	57.9	22.4	41.1
Age at baseline (years)	50.7 (±8.5)	51.2 (±8.4)	50.6 (±8.7)	49.9 (±8.5)	50.6 (±8.6)	55.7 (±6.7)	56.3 (±7.3)
25(OH)D (nmol/L)^†^	27.3 (±6.3)	40.4 (±2.9)	50.8 (±3.3)	70.3 (±16.9)	47.2 (±18.3)	44.6 (±18.9)	44.6 (±18.3)
Season of blood draw							
Jan–Mar	25.9	29.8	26.1	21.0	25.7	23.2	28.5
Apr-Jun	21.6	26.8	23.5	24.6	24.1	21.5	22.6
Jul–Sept	26.8	22.2	27.0	27.8	25.9	28.3	24.9
Oct–Sept	25.7	21.2	23.4	26.6	24.3	27.0	24.0
BMI categories							
Normal weight	43.0	37.3	46.3	52.7	44.8	22.2	32.7
Overweight	34.5	42.4	40.2	38.5	38.9	51.3	47.0
Obese	22.5	20.3	13.5	8.8	16.3	26.5	21.3
Waist circumference (cm)							
Women	82.9 (±13.1)	82.6 (±12.2)	79.2 (±9.9)	77.1 (±9.1)	80.6 (±11.5)	86.5 (±12.5)	84.5 (±12.0)
Men	96.5 (±11.2)	96.9 (±10.7)	94.8 (±9.9)	93.1 (±8.8)	95.2 (±10.2)	98.9 (±9.7)	97.5 (±10.8)
Alcohol intake (g/day)	14.8 (±22.6)	14.6 (±16.8)	15.3 (±17.1)	19.0 (±21.0)	15.9 (±19.6)	16.8 (±19.7)	20.1 (±26.4)
Smoking status							
Never	47.8	46.9	46.9	45.4	46.8	29.2	37.3
Former	25.2	31.9	35.7	38.1	32.7	30.6	34.2
Current	27.0	21.2	17.4	16.5	20.5	40.2	28.5
Physical activity index							
Inactive	21.4	20.3	13.7	12.8	17.0	22.9	25.5
Moderately Inactive	38.3	35.5	38.6	36.2	37.2	36.9	35.7
Moderately Active	25.7	25.5	25.0	28.3	26.1	23.6	20.2
Active	14.6	18.7	22.7	22.7	19.7	16.6	18.6
Education level							
Low	24.0	21.8	21.6	20.8	22.1	34.0	33.5
Medium	44.5	44.1	40.9	44.7	43.5	36.3	39.3
High	31.5	34.1	37.5	34.5	34.4	29.7	27.2
Prevalent hypertension^‡^	32.8	35.5	32.1	27.4	31.9	48.4	52.1
Prevalent diabetes^‡^	5.6	4.9	3.0	2.4	4.0	14.5	13.9

* Includes 20 incident cases of MI and 27 incident cases of stroke;

† Season-standardized;

‡ Self-reported;

Presented values are percentages. Means (standard deviations) are shown for age, 25(OH)D, waist circumference, and alcohol intake;

In the subcohort, there were more women in the lowest 25(OH)D quartile compared to the overall percentage of women. Age at baseline was similar across 25(OH)D quartiles. The proportions of overweight and obese subjects, current smokers, physically inactive subjects, and subjects with prevalent hypertension or diabetes were lower at higher 25(OH)D levels, while average alcohol intakes increased across 25(OH)D quartiles. There was no clear relation between education levels and 25(OH)D levels. Mean plasma 25(OH)D levels were similar among participants from Heidelberg (47.4 nmol/L, ±19.6) and Potsdam (47.0 nmol/L, ±17.2) (p=0.63).

### Associations between plasma 25(OH)D and incident myocardial infarction and stroke

Prospective associations between plasma 25(OH)D levels and the risks of MI and stroke are shown in Table 2. In the basic model adjusted for sex and BMI, 25(OH)D was significantly inversely associated with the risk of MI (HR between quartile 1 and quartile 4 [95% confidence interval]: 1.43 [1.07-1.92], p_linear trend_<0.01). After additional adjustment for waist circumference, alcohol intake, physical activity, smoking, and education levels, the association attenuated and was no longer significant (HR [95% CI]: 1.17 [0.86-1.58], p_linear trend_=0.19). Concerning incident stroke, the basic model revealed a significant association with 25(OH)D levels (HR [95% CI]: 1.37 [1.02-1.84], p_linear trend_=0.05). This association also attenuated by multiple adjustment (HR [95% CI]: 1.25 [0.92-1.70], p_linear trend_=0.19). Similarly, 25(OH)D levels were significantly inversely related to CVD risk in the basic model (HR [95% CI]: 1.41 [1.11-1.79], p_linear trend_<0.01), but not in the multivariable model (HR [95% CI]: 1.19 [0.93-1.52], p_linear trend_=0.12) when using MI and stroke as a composite endpoint (Table 2).

**Table 2:** Hazard ratios for cardiovascular diseases according to quartiles of 25(OH)D.

**25(OH)D quartile**	**Cases**		**Model 1**			**Model 2**		
(Median nmol/L)*	**N**	**(%)**	**HR**	**95% CI**	**P trend^†^**	**HR**	**95% CI**	**P trend^†^**
Myocardial Infarction								
Q4 (66.5)	118	(21.1)	1.0	(Referent)		1.0	(Referent)	
Q3 (50.5)	117	(20.9)	0.95	(0.70-1.28)		0.85	(0.62-1.17)	
Q2 (40.4)	158	(28.3)	1.24	(0.93-1.66)		1.07	(0.78-1.45)	
Q1 (28.9)	166	(29.7)	1.43	(1.07-1.92)	<0.01	1.17	(0.86-1.58)	0.19
Stroke								
Q4 (66.6)	111	(23.6)	1.0	(Referent)		1.0	(Referent)	
Q3 (50.5)	101	(21.4)	0.86	(0.63-1.17)		0.83	(0.60-1.13)	
Q2 (40.4)	102	(21.7)	0.83	(0.61-1.12)		0.83	(0.61-1.14)	
Q1 (28.9)	157	(33.3)	1.37	(1.02-1.84)	0.05	1.25	(0.92-1.70)	0.19
CVD as composite endpoint								
Q4 (66.6)	229	(22.2)	1.0	(Referent)		1.0	(Referent)	
Q3 (50.5)	218	(21.2)	0.89	(0.70-1.14)		0.84	(0.65-1.09)	
Q2 (40.4)	260	(25.2)	1.06	(0.83-1.35)		0.96	(0.75-1.24)	
Q1 (28.9)	323	(31.4)	1.41	(1.11-1.79)	<0.01	1.19	(0.93-1.52)	0.12

Hazard Ratios calculated by Cox regression analyses using Prentice weights to account for the case-cohort design;

Model 1 adjusted for BMI and sex, stratified by center and age at baseline;

Model 2 additionally adjusted for waist circumference, alcohol intake, education level, physical activity, and smoking

* Quartile medians from the subcohort;

† P for trend calculated modeling quartile medians as continuous variable;

**Figure 3:**
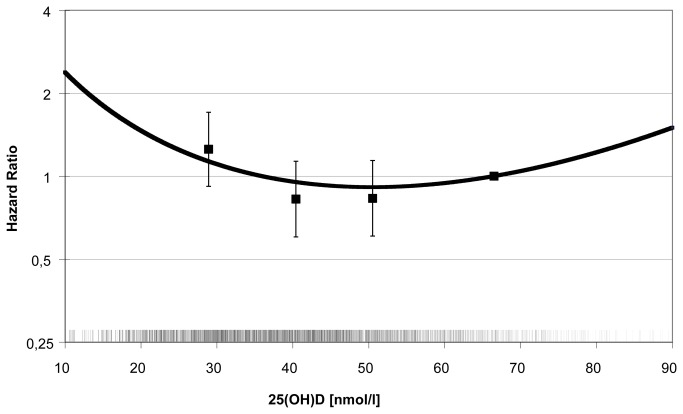
Nonlinear association between plasma 25(OH)D and incident stroke. The curve represents the following second degree fractional polynomial function of 25(OH)D that is significantly (p<0.01) associated with incident stroke after multivariable adjustment: *HR*_*stroke*_ *= exp*[((x)^*1*^ *×*(*-0.19457*) *+* (x)^*1*^ *×*(*0.03954*)*×log*(*x*)) *–* ((66.5)^*1*^ *×*(*-0.19457*) *+* (66.5)^*1*^ *×*(*0*. *03954*)*×log*(*x*))]*;* Additionally, hazard ratios of stroke by 25(OH)D quartiles from the fully adjusted model are presented. The reference for the polynomial function was set at 66.5 nmol/L, the median value in the highest 25(OH)D quartile. The short vertical ticks above the horizontal axis represent the distribution of season-standardized 25(OH)D values in incident stroke cases and the subcohort.

With regard to MI and total CVD, there was no indication for a nonlinear relation with 25(OH)D using fractional polynomials. By contrast, there was a significant (p<0.01) nonlinear association between 25(OH)D and incident stroke characterized by a j-shaped curve after transforming 25(OH)D levels to a second degree polynomial (Figure 3). A categorization by clinical cut-points revealed an increased risk of stroke in participants with deficient 25(OH)D levels (<25 nmol/L) as compared to participants with levels above 50 nmol/L (HR [95% CI]: 1.54 [1.05-2.27], p=0.03, Table 3). The same was true concerning MI risk (HR [95% CI]: 1.56 [1.08-2.25], p=0.02) and CVD risk (HR [95% CI]: 1.53 [1.12-2.09], p<0.01). The risks of both MI and stroke in subjects with 25(OH)D levels between 25 and 49.9 nmol/L did not differ significantly from those of subjects with levels of ≥50 nmol/L.

**Table 3:** Hazard ratios for cardiovascular diseases according to clinical vitamin D status categories.

**Clinical category**	**25(OH)D (nmol/L)**	**N cases/subco.**	**Model 1**			**Model2**		
			**HR**	**95% CI**	**P**	**HR**	**95% CI**	**P**
Myocardial Infarction								
Sufficiency	≥50	183/802	1.0	(Referent)				
Mild deficiency	25-49.9	315/1162	1.24	(0.99-1.55)	0.06	1.10	(0.87-1.39)	0.43
Moderate to severe deficiency	<25	61/168	1.81	(1.25-2.64)	<0.01	1.56	(1.08-2.25)	0.02
Stroke								
Sufficiency	≥50	171/802	1.0	(Referent)				
Mild deficiency	25-49.9	240/1162	0.99	(0.79-1.24)	0.93	0.95	(0.75-1.19)	0.65
Moderate to severe deficiency	<25	60/168	1.81	(1.25-2.62)	<0.01	1.54	(1.05-2.27)	0.03
CVD (composite)								
Sufficiency	≥50	354/802	1.0	(Referent)		1.0	(Referent)	
Mild deficiency	25-49.9	555/1162	1.11	(0.93-1.33)	0.25	1.01	(0.84-1.22)	0.90
Moderate to severe deficiency	<25	121/168	1.80	(1.33-2.45)	<0.01	1.53	(1.12-2.09)	<0.01

Hazard ratios calculated by Cox regression analyses using Prentice weights to account for the case-cohort design;

Model 1 adjusted for BMI and sex, stratified by center and age at baseline;

Model 2 additionally adjusted for waist circumference, alcohol intake, education level, physical activity, and smoking

Additional adjustment for self-reported prevalent hypertension, dyslipidemia, diabetes, drug use (anti-hypertensive drugs, NSAIDS, glucocorticosteroids, bisphosphonates, anti-epileptics, oral contraceptives, or hormone replacement therapy), and general use of vitamin and mineral supplements did not affect the reported associations substantially (data not shown). There was no evidence for effect modification of the association between 25(OH)D and disease risks by other risk variables. Also, subgroup analyses for nonfatal MI, fatal MI, ischemic stroke, and hemorrhagic stroke did not reveal significant associations. Finally, in sensitivity analyses excluding cases that had occurred within the first two years after baseline, 25(OH)D levels were not related to MI or stroke risk (data not shown). Analyses showed similar results when using raw 25(OH)D levels adjusting for month of blood draw or season-specific quartiles (data not shown).

### Associations between SNPs and plasma 25(OH)D

Four SNPs (rs1155563 and rs2282679 in the *GC* locus, as well as rs12785878 and rs3829251 in the *DHCR7/NADSYN1* locus) were significantly associated with season-adjusted 25(OH)D levels in the subcohort (Table 4). The variance in 25(OH)D explained by these four SNPs ranged from 0.7% (rs3829251) to 3.0% (rs2282679). As the two SNP pairs in the *GC* locus (rs1155563 and rs2282679) and the *DHCR7/NADSYN1* locus (rs12785878 and rs3829251) were in high LD (r^2^>0.8), only rs2282679 and rs12785878 were used to build SNP scores. A simple SNP score based on the sum of risk alleles from both SNPs explained 4.4% of the variance in plasma 25(OH)D levels, while a SNP score based on risk alleles weighted by beta coefficients from linear regression models explained 4.2% adjusting for age, sex and center (data not shown). SNP associations with 25(OH)D levels were similar when analyzing data of the entire case-cohort or when using raw 25(OH)D levels adjusting for month of blood draw instead of season-standardized levels (data not shown).

**Table 4:** Associations between candidate SNPs, season-standardized 25(OH)D levels, and the risks cardiovascular diseases.

			**25(OH)D levels by alleles**				**Risk of myocardial Infarction^†^**				**Risk of stroke^†^**				**Risk of CVD (composite)^†^**			
SNP, locus*	**Allele**	**N subcohort**		**Mean 25(OH)D**	P**	**R^2^**	**N cases**	**HR**	**95% CI**	**P**	**N cases**	**HR**	**95% CI**	**P**	**N cases**	**HR**	**95% CI**	**P**
rs1155563,	T/T	1059		49.7			281	Ref.			243	Ref.			524	Ref.		
*GC*	T/C	832		45.1			224	1.07	(0.86-1.33)		185	0.99	(0.79-1.24)		409	1.05	(0.88-1.25)	
	C/C	164		42.4	<0.01	2.1	36	0.80	(0.53-1.21)	0.73	32	0.90	(0.59-1.37)	0.69	68	0.84	(0.60-1.18)	0.72
rs2282679,	A/A	1058		50.0			288	Ref.			249	Ref.			537	Ref.		
*GC*	A/C	827		45.0			216	0.97	(0.77-1.21)		183	0.91	(0.73-1.14)		399	0.94	(0.79-1.13)	
	C/C	166		40.3	<0.01	3.0	38	0.85	(0.57-1.27)	0.48	31	0.85	(0.56-1.31)	0.32	69	0.85	(0.61-1.19)	0.31
rs3829251,	G/G	1415		48.2			380	Ref.			314	Ref.			694	Ref.		
*DHCR7/*	G/A	594		45.1			150	1.04	(0.83-1.31)		140	1.15	(0.91-1.45)		290	1.09	(0.90-1.31)	
*NADSYN1*	A/A	60		44.0	<0.01	0.7	15	1.17	(0.63-2.17)	0.61	11	1.0	(0.50-1.98)	0.34	26	1.09	(0.65-1.83)	0.38
rs12785878,	T/T	1081		49.0			290	Ref.			241	Ref.			531	Ref.		
*DHCR7/*	T/G	806		46.2			210	1.04	(0.84-1.29)		181	1.05	(0.84-1.31)		391	1.06	(0.89-1.27)	
*NADSYN1*	G/G	172		40.3	<0.01	1.7	42	1.12	(0.76-1.65)	0.55	42	1.23	(0.83-1.81)	0.35	84	1.20	(0.87-1.65)	0.38
rs6599638,	G/G	555		46.3			135	Ref.			127	Ref.			262	Ref.		
*C10orff88*	G/A	1020		47.6			268	1.10	(0.86-1.41)		231	1.02	(0.79-1.31)		499	1.07	(0.87-1.31)	
	A/A	488		47.1	1.0	0.0	141	1.14	(0.85-1.52)	0.36	106	0.92	(0.68-1.23)	0.56	247	1.05	(0.83-1.33)	0.70
rs10741657,	G/G	768		46.2			208	Ref.			183	Ref.			391	Ref.		
*CYP2R1*	G/A	966		47.0			251	1.07	(0.86-1.34)		212	1.03	(0.82-1.30)		463	1.06	(0.88-1.28)	
	A/A	317		49.2	0.10	0.3	87	0.94	(0.69-1.28)	0.87	66	0.81	(0.60-1.24)	0.33	153	0.90	(0.69-1.16)	0.62
rs10877012,	G/G	919		48.1			243	Ref.			213	Ref.			456	Ref.		
*CYP27B1*	T/G	910		46.7			251	1.10	(0.89-1.37)		203	1.00	(0.80-1.25)		454	1.11	(0.89-1.40)	
	T/T	238		45.3	0.16	0.3	49	0.79	(0.55-1.13)	0.59	47	0.86	(0.59-1.23)	0.53	96	1.07	(0.86-1.35)	0.38
rs6013897,	T/T	1315		47.7			337	Ref.			276	Ref.			613	Ref.		
*CYP24A1*	T/A	680		46.3			185	1.07	(0.86-1.34)		164	1.16	(0.92-1.45)		349	1.09	(0.90-1.31)	
	A/A	67		46.5	0.99	0.1	27	1.50	(0.90-2.52)	0.18	22	1.54	(0.90-2.63)	0.07	49	1.48	(0.96-2.30)	0.07
SNP Score^‡^	Risk alleles																	
	0	546		52.4			159	Ref.			127	Ref.			286	Ref.		
	1	865		47.3			210	0.88	(0.69-1.13)		190	1.00	(0.77-1.29)		400	0.94	(0.76-1.13)	
	2	462		43.9			123	0.93	(0.70-1.25)		113	1.05	(0.78-1.42)		236	1.01	(0.79-1.29)	
	≥3	161		38.9	<0.01	4.4	41	1.07	(0.71-1.62)	1.0	30	0.90	(0.57-1.42)	0.95	71	1.00	(0.71-1.42)	0.90

* Percentages of missing values for single SNPs: rs1155563: 3.5%, rs2282679:3.4%, rs3829251:2.8%, rs6013897: 2.9%, rs6599638:3.0%, rs10741657:3.4%, rs10877012:3.0%, and rs12785878:3.3%;

** P for linear trend calculated by ANCOVA within Generalized Linear Models modeling the number of risk alleles as continuous score, adjusted for age, sex and study center; Initial p values were multiplied by 8 to account for the number of tested SNPs;

† Calculated by Cox regression analyses stratified by age and center, and adjusted for sex; P for linear trend calculated modeling the number of risk alleles as continuous score; P values from Cox regressions were not corrected for multiple testing because all associations were non-significant;

‡ Consisting of rs2282679 and rs12785878; rs1155563 and rs2282679 in the *GC* locus, as well as rs3829251 and rs12785878 in the *DHCR7/NADSYN1* loci were in strong linkage disequilibrium (R^2^>0.8);

No deviation from Hardy-Weinberg-Equilibrium was detected for any of the SNPs

### Associations between SNPs and incident cardiovascular diseases

Associations between genetic 25(OH)D determinants and incident MI and stroke are shown in Table 4. None of the SNPs was significantly related to the risks of MI or stroke in the co-dominant model, irrespective of the variance in 25(OH)D explained. Further, the selected SNPs were not significantly associated with incident CVD, i.e. stroke and MI as a composite endpoint. There were no significant linear trends for associations between the SNPs and incident MI, stroke, or CVD when using SNPs as continuous variables in additive models, even without correcting p-values for multiple testing. With respect to the SNP score, Cox regression revealed no significant association with the risk of MI (HR of subjects with ≥3 risk alleles compared to subjects with none [95% CI]: 1.07 [0.71-1.62], p_linear trend_=1.0). The same was true regarding incident stroke (HR [95% CI]: 0.90 [0.57-1.42], p_linear trend_=0.95). Finally, there was no significant association between the SNP score and incident total CVD (HR [95% CI]: 1.0 [0.71-1.42], p_linear trend_=0.90). Modeling circulating 25(OH)D as continuous score by steps of 13.5 nmol/L, i.e. the difference in 25(OH)D between extreme categories of our SNP score, yielded no significant associations concerning incident MI (HR [95% CI]: 0.92 [0.84-1.01], p_linear trend_=0.07) and stroke (HR [95% CI]: 0.95 [0.86-1.04], p_linear trend_=0.26), while a non-significant trend for an inverse association was observed concerning overall CVD (HR [95% CI]: 0.93 [0.87-1.002], p_linear trend_=0.06) in fully adjusted Cox models.

Regression models on SNPs and CVD endpoints including prevalent cases of MI and stroke that had been excluded from analyses on circulating 25(OH)D and incident CVD did not reveal results that differed substantially from those presented in Table 4 (data not shown). Dominant or recessive models showed no significant associations for any of the SNPs (data not shown). Lastly, allele scores including additional SNPs or alleles weighted by beta coefficients were not related to incident MI, stroke, or CVD (data not shown).

## Discussion

In the present study, we found no significant linear associations between 25(OH)D levels and the risks of stroke and MI, although we did observe increased risks of both diseases at 25(OH)D levels below 25 nmol/L. Four out of eight candidate SNPs were significantly associated with 25(OH)D levels. However, neither single SNPs nor allele scores were related to the risks of MI or stroke. Thus, our results provide no support for a major causal role of vitamin D in the development of MI and stroke.

With regard to the lack of linear associations between vitamin D status and the risks of MI and stroke in our study two aspects are worth noting. First, plasma 25(OH)D levels in our population were relatively low compared to other populations worldwide [32]. Only few subjects in our study had 25(OH)D values above 75 nmol/L (5.8% in the subcohort) that are considered optimal regarding CVD prevention by some researchers [33]. Second, the majority of incident cases in our study were nonfatal. Notably, a large prospective study from Denmark has recently shown that 25(OH)D levels were associated with incident fatal MI, but not nonfatal MI [10]. The authors discussed that their finding supported the notion that 25(OH)D may rather be a marker of overall health than a causal factor in CVD etiology. In the Health Professionals Study, the association with 25(OH)D was also stronger for fatal CHD than for nonfatal MI [34], and a similar tendency was observed in the MIDSPAN study [35]. Hence, the hypothesis that 25(OH)D may be related to fatal MI rather than nonfatal MI deserves further study, even though the Intermountain study and the Cardiovascular Health Study 25(OH)D did show associations between 25(OH)D and the risks of incident nonfatal MI [36,37]. Nonetheless, the observation that inverse associations detected in our raw models attenuated after adjusting for classical CVD risk factors may imply that low 25(OH)D is a marker of an adverse CVD risk profile as well.

Interestingly, associations between 25(OH)D and incident MI have been assessed only recently in the MONICA/KORA-Augsburg study that resembled our study regarding the evaluation concept and the population [38]. In agreement with our results, 25(OH)D levels were not related to incident MI in men, whereas a significant inverse association that remained stable after rigorous adjustment was observed in women. No such association restricted to female participants was evident in the EPIC-Germany CVD case-cohort study. However, it should be noted that the numbers of incident cases in women were rather low in both studies and that a possible gender difference concerning 25(OH)D and the risk of MI therefore requires further investigation.

The SNP selection in the present study appears to be in line with recent suggestions for genetic markers in Mendelian Randomization studies on vitamin D [16,20,39,40]. As opposed to us, rs4588 in the *GC* locus instead of rs2282679 was used in the 1958 British birth cohort study [16]. However, both SNPs were in strong LD so that similar results using rs4588 instead of rs2282679 in our study can be assumed. Likewise, rs2298850 was used instead of rs2282679 in the Tromsø Study. Yet, both SNPs were in high LD and between allele-differences in 25(OH)D were similar [20]. In the *DHCR7/NADSYN1* locus, rs3794060 was used in the Tromsø Study and reported to be in perfect LD with rs12785878, the SNP that we chose [20]. In a recent GWAS based on 5 cohorts, rs2282679, rs12785878, rs3829251, and rs6013897 were identified as the best predictors of circulating 25(OH)D [39]. Interestingly, it appeared that the explanatory power with respect to circulating 25(OH)D was better when using a score out of these SNPs as compared to polygenic scores consisting of several thousand SNPs [39]. Also, the variance explained by the two strongest SNPs was similar to the variance explained by the listed four candidate SNPs [39]. These observations are in line with our finding that adding further SNPs to an allele score consisting of rs2282679 and rs12785878 did not increase the explained variance in 25(OH)D.

Although we selected the most relevant SNPs, the variance in plasma 25(OH)D explained by our SNP score did not exceed 4.4%. This modest proportion of explained variance in 25(OH)D levels may have contributed to our failure to detect significant associations between SNPs and the risks of MI and stroke. Nevertheless, the average difference in 25(OH)D between extreme categories of our SNP score was 13.5 nmol/L. Considering the increased risks of stroke and MI at 25(OH)D levels below 25 nmol/L in our study, it does not seem implausible that even a smaller fraction of genetically determined 25(OH)D may affect CVD risk. That said, the limited sample size of our study did not allow for separate analyses of associations between SNPs and CVD risk in subjects with insufficient 25(OH)D levels.

Thus far, two observational studies have reported on genetic determinants of circulating 25(OH)D in relation to CVD risk. The Tromsø Study included some 2000 incident MI cases [20], and, like our study, did not show associations between SNPs and MI risk, the HR of MI being 0.93 (95% CI: 0.82–1.06) for subjects in the highest quartile of a genotype score based on rs2298850 (*GC*), rs10741657 (*CYP2R1*), rs3794060 (*DHCR7/NADSYN1*), and rs6013897 (*CYP24A1*). Also, no association between genetic vitamin D status determinants (rs2282679, rs12785878, and rs10741657) and CVD mortality was observed in a population of 3316 subjects scheduled for coronary angiography comprising 619 later cases of fatal CVD [41]. Still, we cannot rule out the detection of modest associations between SNPs related to vitamin D status and MI or stroke in larger consortia, as a post hoc calculation revealed that we only had the power of 0.62, 0.46 and 0.42 to detect HRs of 1.3 for incident overall CVD, MI, and stroke, respectively, between extreme categories of our SNP score. Pooling data from observational studies in larger Mendelian Randomization projects may help overcome such limitations of statistical power.

Besides the sample size, a further limitation of our study was that we could assess 25(OH)D levels only at a single occasion. However, despite the seasonal variation in 25(OH)D, several large studies have shown acceptable long-term correlations between repeated 25(OH)D measurements that are comparable to those of other biomarkers of CVD [42-45]. Moreover, we did not have the opportunity to measure levels of the vitamin D binding protein or 1,25(OH)_2_D. Vitamin D binding protein levels would have been of particular interest, as the strongest genetic determinants of 25(OH)D in GWAS and in our study were from the *GC* gene. Of note, recent prospective studies on cancer suggest that circulating levels of the vitamin D binding protein may crucially affect associations between 25(OH)D and disease risks by determining the free fraction of 25(OH)D [46-49]. Finally, we could not adjust for further potential confounders, such as blood lipids, blood pressure, or CRP.

In summary, we did not detect clear linear inverse relationships between 25(OH)D and CVD risk. The attenuation of the associations between 25(OH)D and CVD risks by adjustment for classical CVD risk factors in our study support the notion of 25(OH)D being a global health status marker rather than an independent causal factor in CVD etiology. The lack of associations between genetic vitamin D status determinants and CVD risk suggests that associations between low vitamin D levels and CVD risk may be due to uncontrolled confounding or reverse causation. However, a detection of genetic effects related to vitamin D status on CVD risk remains possible, and our results on genetic determinants of 25(OH)D require a cautious interpretation due to limited statistical power. The results of large consortia applying the Mendelian Randomization concept as well as upcoming RCTs will facilitate an evidence-based appraisal of the role of vitamin D with respect to CVD risk.
